# Successful control of intractable Impella 5.5 graft site bleeding using a Viabahn stent graft: A case report

**DOI:** 10.1016/j.jccase.2026.05.016

**Published:** 2026-06-19

**Authors:** Seimei Kure, Taiichi Takasaki, Khoirur Rijal Ashsholih, Toshifumi Hiraoka, Shinya Takahashi

**Affiliations:** Department of Cardiovascular Surgery, Hiroshima University Hospital, Hiroshima, Japan

**Keywords:** Impella 5.5, Viabahn stent graft, Bleeding

## Abstract

Impella 5.5 (Abiomed Inc., Danvers, MA, USA) provides effective mechanical circulatory support in severe heart failure but requires surgical implantation via a prosthetic graft, which may be complicated by bleeding. We report a case of uncontrollable oozing hemorrhage at the axillary graft anastomosis site during Impella 5.5 support in a 35-year-old man with acute decompensated heart failure due to idiopathic cardiomyopathy. The patient had previously been supported with Impella CP, resulting in severe coagulopathy. To control the bleeding while preserving limb perfusion, a Viabahn covered stent (Vascutek Ltd, Inchinnan, United Kingdom) was deployed in the right subclavian artery to cover the graft anastomosis site, achieving immediate and complete hemostasis. After subsequent recovery of cardiac function, the Impella 5.5 was safely explanted. Viabahn stent graft placement may be an effective rescue strategy for intractable graft site bleeding during Impella 5.5 support when standard surgical hemostasis fails, allowing continuation of mechanical support and safe device removal.

**Learning objective:**

Impella 5.5–related graft-site bleeding associated with coagulopathy after Impella CP or extracorporeal membrane oxygenation can be effectively controlled by Viabahn stent graft placement when conventional surgical hemostasis fails.

## Introduction

Impella (Abiomed Inc., Danvers, MA, USA) is widely used for mechanical circulatory support in acute decompensated heart failure. Impella CP can be inserted percutaneously via the femoral artery, whereas Impella 5.5 requires surgical insertion, typically via the axillary artery using a prosthetic graft. When Impella 5.5 is implanted, it is often done after Impella CP or extracorporeal membrane oxygenation (ECMO) has already been in use. As a result, hemostasis at the prosthetic graft anastomosis can be challenging because of a bleeding tendency due to consumption of coagulation factors and increased tissue fragility caused by tissue edema.

## Case report

A previously healthy 35-year-old man developed idiopathic cardiomyopathy with a left ventricular ejection fraction of 15% and was transferred to our hospital under endotracheal intubation. After admission, an Impella CP was inserted for left ventricular support. Because activated clotting time (ACT) and activated partial thromboplastin time (APTT) were discrepant, heparin was adjusted to maintain an APTT of 40–60 s rather than targeting ACT. Native lung oxygenation was adequate, and ECMO was not required. The Impella CP was operated at P9, and complications such as hemolysis were observed. In addition, the patient developed fever and elevated inflammatory markers, suggesting a septic state, and subsequently exhibited a bleeding tendency. As subsequent recovery of left ventricular function was limited, the device was upgraded to an Impella 5.5 on hospital day 6.

The right axillary artery measured 6.3 mm, allowing passage of the Impella 5.5. A 10-mm Gelsoft graft (Vascutek Ltd, Inchinnan, United Kingdom) was anastomosed to the right axillary artery. The Impella CP was removed, and an Impella 5.5 was implanted through the graft. During surgery, the vascular wall was fragile, and persistent bleeding from needle holes was observed due to coagulopathy. Hemostasis was eventually achieved with additional sutures and fibrin glue, and the patient was transferred back to the intensive care unit (ICU). Despite the bleeding tendency, a bicarbonate-based purge solution was not approved by our institutional ethics committee; therefore, a standard heparinized purge solution was used.

After ICU admission, continuous bleeding was noted from the right axillary surgical site, accompanied by further deterioration of coagulation parameters. Significant postoperative coagulopathy developed, with a prothrombin time and international normalized ratio (PT-INR) of 8.7, unmeasurable APTT, thrombocytopenia (platelet count 83,000/μL). After transfusion, PT-INR improved to 1.8, but APTT remained >180 s. We therefore determined that hemostasis could not be achieved with transfusion alone prompting reopening of the surgical site in the ICU for inspection. Oozing was observed from the needle holes of the prosthetic graft, and hemostasis could not be achieved despite additional sutures and topical hemostatic agents.

**Fig. 1:**
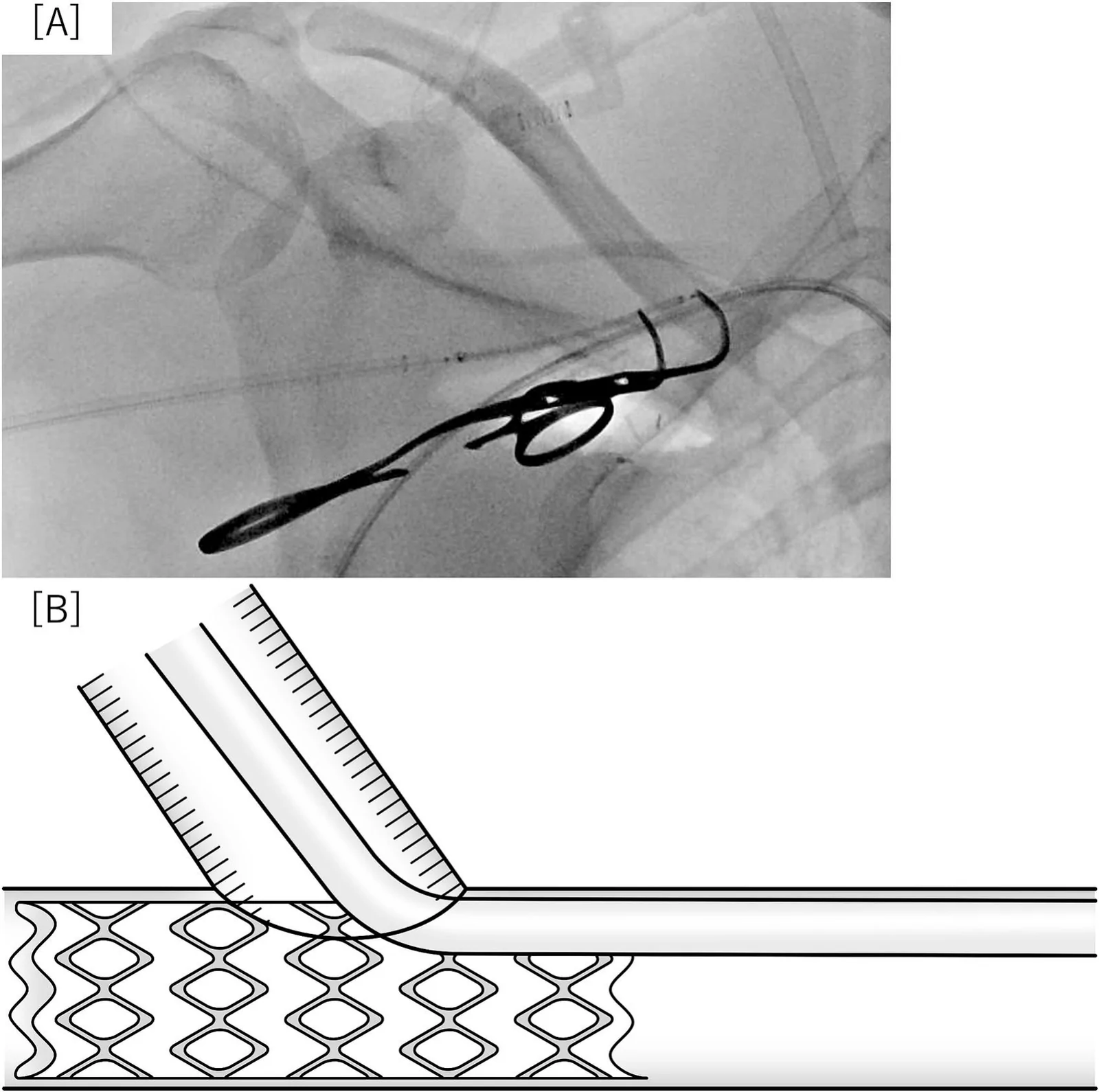
A vascular clamp was placed proximally on the subclavian artery as a fluoroscopic marker (A), and a Viabahn stent graft was deployed distally to cover the graft anastomosis (B).

Because surgical hemostasis failed, endovascular treatment was selected. To preserve limb perfusion and ensure hemostasis, a Viabahn covered stent (7 mm * 5 cm) was planned for the right subclavian artery. Under direct exposure, the right brachial artery was accessed, and a sheath was introduced. Because later stent graft removal might be necessary, a vascular clamp was placed on the proximal side of the subclavian artery and used as a fluoroscopic marker (Fig. 1A), and a Viabahn stent graft was deployed distal to it to cover the prosthetic graft anastomosis (Fig. 1B). As a result, the proximal landing zone extended 17 mm beyond the anastomosis, and no side branches were covered. The placement of the Viabahn successfully achieved bleeding control.

Five days later, after noticeable cardiac function improvement, Impella explantation was planned. Preparations included inserting a Mustang balloon catheter (Boston Scientific Corporation, Minneapolis, MN, USA) via the right brachial artery up to the subclavian segment, ready for proximal occlusion if needed.

**Fig. 2:**
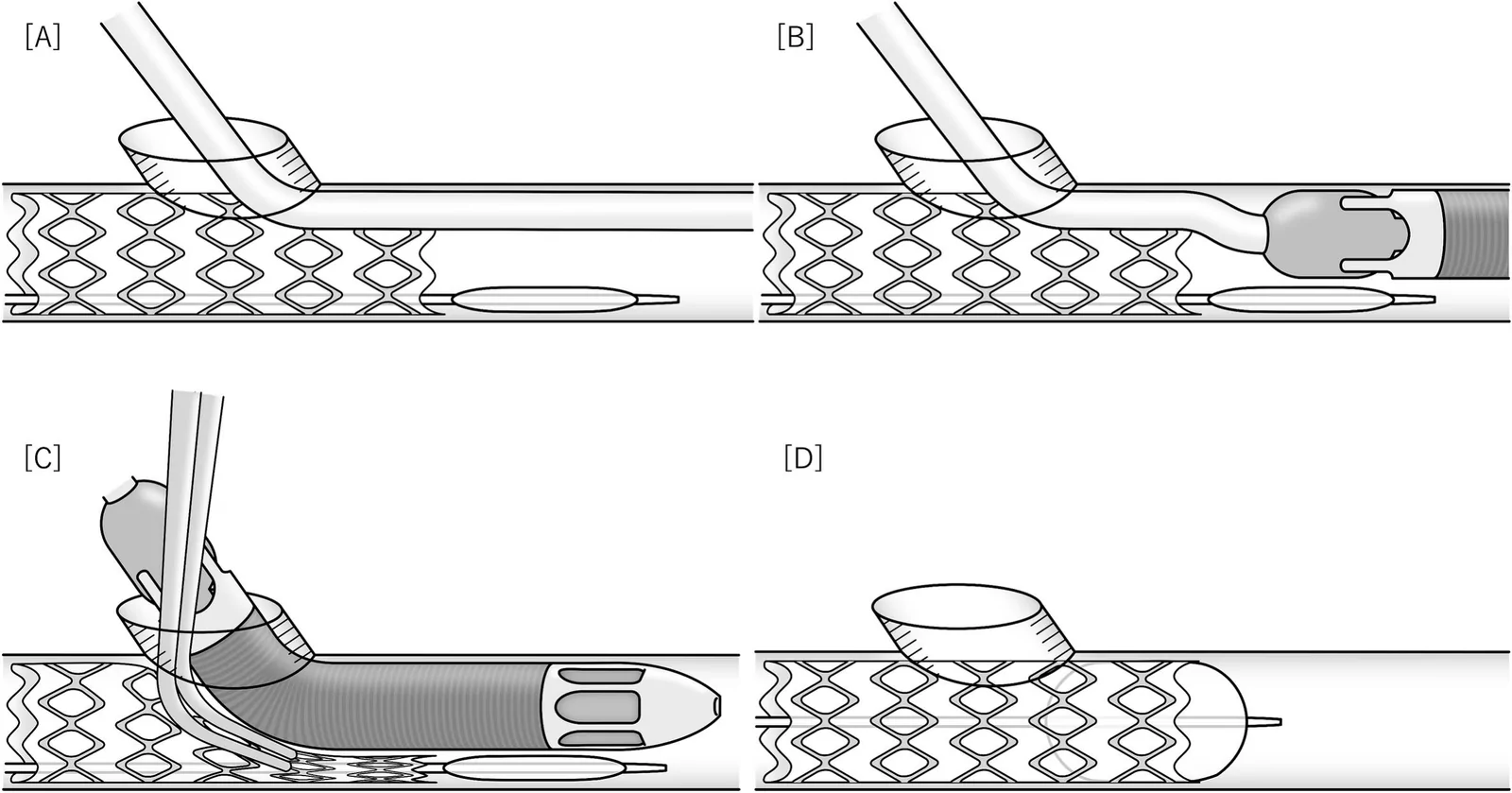
The axillary graft was reopened and divided near the anastomosis, exposing the Viabahn and Impella (A). With Impella at P2, the catheter was withdrawn to the stent edge, and a curved clamp was used to create a ramp for safe extraction (B, C). A Mustang balloon was then reinflated to secure hemostasis (D).

The axillary incision was reopened, and the graft was divided 1 cm from the anastomosis, exposing both the Viabahn and the Impella catheter within (Fig. 2A). With Impella set to P2, the catheter was gently withdrawn until it reached the edge of the Viabahn stent (Fig. 2B). A curved clamp was inserted between the stent and the catheter, compressing the stent and creating an artificial ramp (Fig. 2C). Using suction to maintain visibility, the Impella was carefully guided along the ramp and extracted successfully. Because the Viabahn was self-expanding, bleeding from the gap after device removal was transient, and the use of a vascular clamp was not required. The Mustang balloon was then repositioned and reinflated at the stent site to ensure hemostasis (Fig. 2D). The graft stump and the wound closed conventionally. Although antiplatelet therapy is typically administered for a Viabahn stent graft, anticoagulation was already being used for atrial tachycardia; therefore, considering the bleeding risk, only anticoagulant therapy was continued.

## Discussion

The incidence of hemorrhagic complications in patients supported by Impella devices has been reported at 6.92%, with access site bleeding at 4.09% [1]. In addition to heparin use, it has been suggested that the high shear stress generated by the axial-flow pump of the Impella leads to degradation of plasma von Willebrand factor, thereby contributing to coagulopathy. Prolonged Impella support has been reported to predispose patients to acquire von Willebrand syndrome [2]. Other studies have also shown that the use of a percutaneous ventricular assist device for more than four days is associated with an increased risk of bleeding [3].

Viabahn, a self-expanding covered stent, has been utilized in vascular injuries and trauma, especially in the subclavian artery [4], [5]. However, reports of its use to control bleeding at Impella graft sites are scarce. This case demonstrates the utility of Viabahn as a life-saving hemostatic measure when traditional surgical techniques fail.

This case suggests that Viabahn stent grafting can be a useful rescue option for uncontrollable Impella 5.5 graft-site bleeding when conventional surgical methods fail.

## Limitations

This report has some limitations. First, we did not assess whether Impella repositioning remained feasible after stent deployment; therefore, device manipulation may be restricted while the Viabahn is in place. Second, the artery in this patient had a favorable diameter (6.3 mm) and good elasticity, which likely facilitated safe device removal. This strategy may be less feasible in smaller or diseased arteries.

## Consent statement

Written informed consent was obtained from the patient for publication of this case report, including accompanying images.

## Declaration of competing interest

The authors declare that there is no conflict of interest.
